# The incubation period during the pandemic of COVID-19: a systematic review and meta-analysis

**DOI:** 10.1186/s13643-021-01648-y

**Published:** 2021-04-08

**Authors:** Wafa Dhouib, Jihen Maatoug, Imen Ayouni, Nawel Zammit, Rim Ghammem, Sihem Ben Fredj, Hassen Ghannem

**Affiliations:** Department of Epidemiology and Preventive Medicine, University of Sousse, Sousse, Tunisia

**Keywords:** COVID-19, Infectious disease incubation period, Contact tracing, Coronavirus

## Abstract

**Background:**

The aim of our study was to determine through a systematic review and meta-analysis the incubation period of COVID-19. It was conducted based on the preferred reporting items for systematic reviews and meta-analyses (PRISMA). Criteria for eligibility were all published population-based primary literature in PubMed interface and the Science Direct, dealing with incubation period of COVID-19, written in English, since December 2019 to December 2020. We estimated the mean of the incubation period using meta-analysis, taking into account between-study heterogeneity, and the analysis with moderator variables.

**Results:**

This review included 42 studies done predominantly in China. The mean and median incubation period were of maximum 8 days and 12 days respectively. In various parametric models, the 95th percentiles were in the range 10.3–16 days. The highest 99th percentile would be as long as 20.4 days. Out of the 10 included studies in the meta-analysis, 8 were conducted in China, 1 in Singapore, and 1 in Argentina. The pooled mean incubation period was 6.2 (95% CI 5.4, 7.0) days. The heterogeneity (*I*^2^ 77.1%; *p* < 0.001) was decreased when we included the study quality and the method of calculation used as moderator variables (*I*^2^ 0%). The mean incubation period ranged from 5.2 (95% CI 4.4 to 5.9) to 6.65 days (95% CI 6.0 to 7.2).

**Conclusions:**

This work provides additional evidence of incubation period for COVID-19 and showed that it is prudent not to dismiss the possibility of incubation periods up to 14 days at this stage of the epidemic.

**Supplementary Information:**

The online version contains supplementary material available at 10.1186/s13643-021-01648-y.

## Background

Since December 2019, the world is facing the pandemic of COVID-19. As of December 8, 2020, a total of cumulative confirmed cases were estimated at more than 68 million and 1.5 million cumulative deaths with a case fatality rate of 2.28% [1, 2].

While awaiting a vaccine, massive public health interventions such as social awareness, social distancing, isolation, quarantine, contact tracing, targeted screening, and border controls have been implemented nationally and globally to limit transmissibility and contain the epidemic since late January [3–6]. The incubation period, one of the key epidemiological parameters, is essential to epidemiological case definition, to determine the appropriate duration of quarantine and to estimate the size of the epidemics. Therefore, it was rapidly being studied from incoming case reports as the epidemic continues. Several studies have confirmed that cases are infectious during the asymptomatic period (latency period) prior to onset and that disease transmission may be carried out [7–10]. Up to this point, the quarantine and isolation duration of exposed or suspected cases is set at 14 days, which is the longest incubation time expected based on initial observations of SARS-CoV-2 and similarity to severe acute respiratory syndrome coronavirus (SARS-CoV) and Middle East respiratory syndrome coronavirus (MERS-CoV) [11].

The distribution of the incubation period in most of the literature is either described through a parametric model or its empirical distribution based on the observed incubation period from the contact-tracing data (specific data indicating the time of exposure). However, the contact-tracing data are challenging and expensive to obtain, and their accuracy can be highly influenced by recall bias. Therefore, previous population dynamics studies tend to make assumptions about the distribution of the incubation period without using the observed data called modeling studies, and we are talking here about an estimate of the incubation period.

Furthermore, estimating and standardizing the incubation period of COVID-19 may vary depending on climate [12], on age or the genetic of the individuals, their pathologies, or their treatment like the long-term use of glucocorticoids which might cause atypical infections and a long incubation period [13].

Thus, a specific maximum duration of the incubation period is needed to answer if the 14-day quarantine is sufficient to protect against the spread of the pandemic. In this systematic review and meta-analysis, we tried to identify studies that recruited symptomatic patients, regardless of sex or age diagnosed with COVID-19, and calculated or estimated the incubation period between December 2019 and December 2020.

## Methods

### Criteria for considering studies for this review

#### Types of studies

The protocol for this review was registered with PROSPERO (international prospective register of systematic reviews) under the number CRD42020196347 (https://www.crd.york.ac.uk/prospero/).

This systematic review was conducted based on the preferred reporting items for systematic reviews and meta-analyses (PRISMA) to study the length of incubation period during the COVID-19 pandemic.

Criteria for eligibility were all published population-based primary literature dealing with incubation period of COVID-19, since December 2019. We included full-text publications and excluded all articles not accepted or peer reviewed, not written in English, editorials, perspective, letter to the editor, review, article info, and comments. We only took articles that used RT-PCR for the diagnosis of COVID-19. Since randomized controlled trials (RCTs) do not apply to this topic, only observational studies with no limit on the number of participants were included. There were no limitations given the types of outcome measures: we accepted all documents that presented results even without a statistical parameter of variability.

#### Types of participants

We included individuals with a confirmed diagnosis of COVID-19 regardless of the severity of symptoms or associated comorbidities. There were no age, gender, or ethnicity restrictions. We excluded studies including populations with other coronavirus diseases (severe acute respiratory syndrome (SARS) or Middle East respiratory syndrome (MERS)). We also excluded studies including populations with mixed viral diseases (e.g., COVID-19 plus influenza).

#### Types of outcome measures

The incubation period was defined as the amount of time between the exposure to SARS-CoV-2 and the onset of symptoms [14].

##### Estimated and calculated incubation period

1. Incubation periods were calculated from observed data based on specific dates indicating the time of exposure. Measures of central tendency were ranges, mean, and median with appropriate dispersion parameters (interquartile range (IQR) and standard deviation (SD)).
2. Incubation periods were estimated on incomplete data or imprecise exposure time using several models of distribution such as log-normal, Weibull, and Gamma distribution [15, 16]. They were presented by the mean and its 95% confidence interval (95% CI) with percentiles of the distribution in some studies.

### Search methods for identification of studies

#### Electronic searches

The literature search was developed by WD and verified by a research librarian. It was carried out on Medline via its PubMed interface, through the following documentary query, as of 01/12/2020: (“Infectious Disease Incubation Period”[Mesh]) AND (“COVID-19”[All Fields] OR “COVID-2019”[All Fields] OR “severe acute respiratory syndrome coronavirus 2”[Supplementary Concept] OR “severe acute respiratory syndrome coronavirus 2”[All Fields] OR “2019-nCoV”[All Fields] OR “SARS-CoV-2”[All Fields] OR “2019nCoV”[All Fields] OR ((“Wuhan”[All Fields] AND (“coronavirus”[Mesh Terms] OR “coronavirus”[All Fields])) AND (2019/12[PDAT] OR 2020[PDAT]))). No filter was applied.

The research was also conducted on Science Direct through its advanced research (only the research article using COVID-19 and the incubation period in the title, abstract, or keywords specified by the author). The literature search was completed on 01/12/2020.

### Data collection and analysis

#### Selection of studies and data extraction

The references were managed using the Zotero software. Firstly, and after exclusion of duplicates, all titles and abstracts of publications identified through the initial primary search were single reviewed for relevance.

**Fig. 1:**
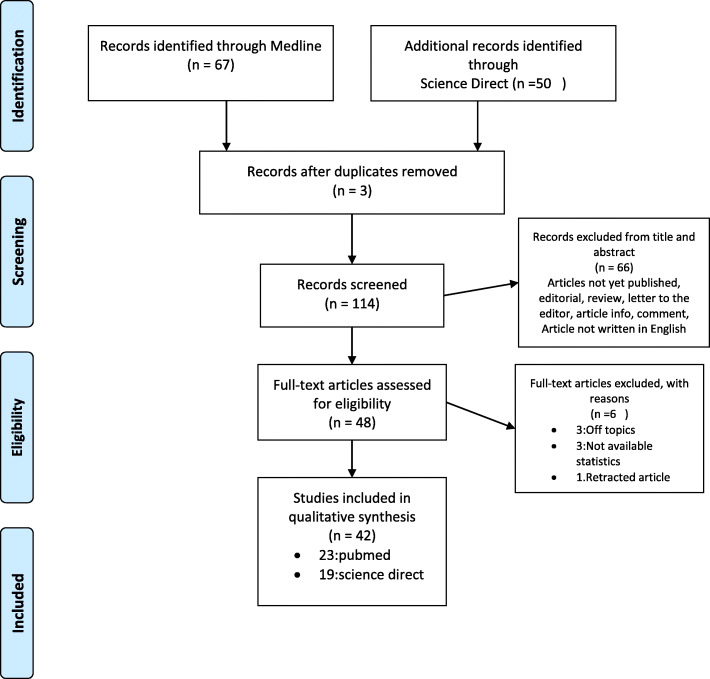
PRISMA study flow diagram for search up to 1 December 2020

Secondly, the final selection of the articles was based on the full texts of papers by retrieving and checking for relevance by two authors (WD, AI) independently of each other, with referral to MJ in the case of discordant opinions. Studies were excluded if they were off topic or if they gave no number or statistics. One article was retracted from Medline during the process of final selection. We documented the study selection process in a flow chart and showed the total numbers of retrieved references and the numbers of included and excluded studies (Fig. 1).

One review author WD performed all data extractions. Two other review authors (AI and MJ) verified the accuracy and the plausibility of extractions.

All authors participated in quality assessment, level of evidence, and grades of recommendations. DW and AI independently reviewed all studies, with disagreements resolved by referral to MJ.

The following bibliometric, epidemiological data were extracted: authors, study design, country or geographical region, period of study, sample size, data and source collection, general characteristics of the studied population (age, sex ratio), exposure history, and duration of incubation period.

The meta-analysis was based on the mean of the distributions either in observed or estimated log-normal distribution data. The meta-analysis included all studies that reported the mean with its SD of the observed incubation period or the mean and corresponding CI of the normal log distribution. Excluded studies where those representing outcome reporting bias. The selection of studies to include in the meta-analysis was conducted by the primary author WD.

#### Assessment of risk of bias

Quality assessment was done according to recommendation of “Quality Assessment Tool for Quantitative Studies” developed in Canada by the Effective Public Health Practice Project (EPHPP) [17]. Once the assessment is fulfilled using a number pre-determined criteria, each examined practice receives a mark ranging between “strong,” “moderate,” and “weak” in three categories (study design, data collection practices, and selection bias). After discussing the ratings and resolving any discrepancy, the global rating for each paper was according to the sum of “weak” ratings given to the three categories (1: strong=0 “weak”; 2: moderate=1 “weak”; and 3: weak=≥2 “weak”) (Additional file 1).

Risk of bias was done according to Chapter 25 of *Cochrane Handbook: assessing risk of bias in a non-randomized study* [: /handbook/current/chapter-25]; assessing risk of bias was presented using Revman 5 tools (Review Manager (RevMan) [Computer program] version 5.3. Copenhagen: The Nordic Cochrane Centre, The Cochrane Collaboration, 2014.).

Level of evidence and grades of recommendations were assessed according to the Scottish Intercollegiate Guidelines Network (SIGN) [18].

Levels of evidence were graded into 8 levels from 1++ (high quality meta-analyses, systematic reviews of randomized controlled trials (RCTs), or RCTs with a very low risk of bias) to 4 (expert opinion).

There were 4 grades of recommendations (A, B, C, and D) based on the results of the level of evidence. D is given if the evidence level was 3 or 4 or extrapolated from studies rated as 2+ [18].

### Data synthesis

A random effects meta-analysis was conducted in the Open Meta Analyst software [19], of the calculated and estimated mean of the log-normal distribution. Forest plots were produced using the same package. Heterogeneity between the studies was assessed using both the *I*^2^ statistic with a cutoff of 50% and the *χ*^2^ test with *P*-value <0.10 and investigated by conducting subgroup analyses of the data set following these moderator variables: population of studies (Chinese or not), severity (hospitalized or not), sex ratio (> or < 1), study quality, and method of calculation (estimated or calculated).

## Results

### Results of the search

We identified 117 records through Medline and Science Direct database searches. After removing 3 duplicates, we screened 114 records based on their titles and abstracts, leaving 48 full manuscripts to be assessed for eligibility (Fig. 1). As a result of this assessment, 42 studies met the inclusion criteria.

### Study characteristics

Over the 42 observational studies, the quality assessment gave 9 strong, 19 moderate, and 14 weak studies (Additional file 1). Most of the studies had a retrospective data collection; 10 had a prospective one [7–9, 20–26]. The sampling methods and sample size recorded varied substantially across studies. In some cases, entire provinces or villages were selected [8, 9, 20, 24–37] whereas in others the focus was on serial cases or family clusters [7, 8, 24, 38–42], hospitals, and laboratory [43–50]. Almost studies were done in China (30 studies), including a study of around 8579 people in 30 provinces [23]. Four studies was conducted in Korea [9, 28, 34, 51], three in Singapore [21, 48, 52], one in France [41], one in Brunei [32], one in Argentina [33], one in Saudi Arabia [53], and two in Germany [37, 42]. The period of all studies was between January and May 2020 (Table 1).

**Table 1:** Characteristics of studies calculating incubation period SARS-COV-2

Authors	Country (province)	Period	Data and source collection	General characteristics of the population study			Exposure history
				*N*	Age^┼┼^ (years)	Sex ratio (M/F)	
Guan et al. [27]	China	11/12/2019 to 29/01/2020	552 hospitals’ medical records	1099	Median =47 IQR [35-58]	1.43	Contact with wildlife Resident or travelers to Wuhan
Ki and Task Force for 2019-nCoV [28]	Korea	20/01/2020 to 10/02/2020	CDC Additional data announced by the press	28	Mean= 42 Range (20–73)	1.15	Close contact with confirmed cases
Chen et al. [43]	China (Chongqing)	28/01/2020 to 11/02/2020	3 hospitals’ medical records	12	Mean=14.5 range (7M–17Y)	1	Resident or travelers to Wuhan Close contact with confirmed cases
Gao et al. [7]	China (Wuxi)	January–March 2020	Data of scientific investigation from “Public Health Emergency Reporting Management Information System”	15	Median= 51 Range (9–74)	1.5	Close contact with confirmed cases
Huang et al. [8]	China (Anhui)	January–February 2020	Information from patients and contacts.	17	Median=22 Range (16–23)	0.75	Close Contact with confirmed cases
Pung et al. [38]	Singapore	February 2020	The ministry of health (first three clusters)	36	- -	-	Close contact with a tourist group from China Public place (company conference/church)
Song et al. [39]	China (Beijing)	16/01/2020 to 29/01/2020	1 hospital’s medical records (4 families)	24	Range (9M–86 Y)	0.37	Close contact with confirmed cases
Tian et al. [44]	China (Beijing)	20/01/2020 to 10/02/2020	57 hospitals’ medical records	262	Median =47.5 Range (6M–94 Y )	0.94	Wuhan travel Close contact with confirmed cases Family cluster cases
Wang et al. [25]	China (Jiangsu )	22/01/2020 to 18/02/2020	Websites of bureau of health and the people’s government.	631	- -	>1	Resident or travelers to Hubei province Close contact with confirmed cases Others
Xia et al. [40]	China (Chongqing)	23/01/2020 to 18/02/2020	Hospital electronic medical record system of patient with severe acute respiratory syndrome	10	Mean =56.5±11.16	1.5	Close contact with confirmed cases
Xu et al. [54]	China (Changzhou)	23/01/2020 to 18/02/2020	Laboratory-confirmed cases	51	Mean =35.0 Range (29–51)	0.96	Resident or travelers to Wuhan Close contact with confirmed or suspected cases
Bernard et al. [41]	France	24/01/2020 to 12/02/2020	Unspecified	3	-	-	-
Yu et al. [30]	China (Shanghai)	As of February 19th, 2020.	CDC	333	Median =50	1.06	Cases with a travel history in Wuhan Close contact with confirmed cases
Li et al. [20]	China (Wuhan)	December 2019 to 22 January 2020	Laboratory-confirmed cases of infected pneumonia(NCIP)	425	Median= 59 Range(15 to 89)	1.29	Contact with wildlife Close contact with suspected cases
Zhang et al. [23]	China outside Hubei	19/01/2020 to 17/02/ 2020	Laboratory-confirmed cases	8579	Median= 44 Range (33–56)	-	Contact with wildlife Close contact with confirmed or suspected cases Resident or travelers to Wuhan
Linton et al. [29]	China	December 2019 to January 2020	Official reports from governmental institutes	158	Most (30–59)	0.58	Resident or travelers to Wuhan
Backer et al. [55]	China	20 to 28 January 2020	CDC	88	Range (2–72)	1.84	Travelers to Wuhan
Lauer et al. [56]	China 24 countries outside China	04/01/2020 to 24/02/2020	Public health reports and news	181	Median= 44.5 IQR [34.0–55.5]	1.56	Resident or travelers to Wuhan Close contact with confirmed cases or travelers from Hubei
Wang et al. [57]	China (Henan)	21/01/2020 to 14/02/2020	CDC	1212	Most (21–60)	1.22	Travelers to Wuhan Close contact with confirmed cases
Bi et al. [24]	China (Shenzhen)	14/01/2020 to 12/02/2020	CDC	3911	Mean=45	0.91	Travelers to Wuhan Close contact with confirmed cases
Zheng et al. [45]	China (Hubei)	16/01/2020 to 04/02/2020	Taihe Hospital medical records	73	Mean=43	1.73	Huanan China Seafood Market 12 cases had no exposure history Family cluster cases
Zhao et al. [46]	China (Jiangsu )	16/01/2020 to 17/02/2020	Jiangsu Hospital medical records	136	Median=49 IQR [33-63]	1	Resident or travelers to Wuhan Close contact with confirmed cases Family cluster cases 14 cases had no exposure history
Zhang et al. [59]	China (Hubei)	22/01/2020 to 28/02/2020	Huanggang Hospital Shandong First Medical University	194	Median=48.3	1.25	Contact with wildlife Resident or travelers to Wuhan
Yang et al. [26]	China (Hubei)	20/01/2020 to 29/02/2020	CDC	672	Range (35–64)	1.08	Wuhan-imported Close contact of imported cases Locally infected
Xiao et al. [31]	China (Hubei, Qinghai, Tibet)	As of February 21th, 2020.	CDC	4741	Median =50	1.06	Resident or travelers to Wuhan Local community transmission
Wong et al. [32]	Brunei	09/03/2020 to 05/04/2020	CDC	135	Median= 36 Range(0.5–72)	1.54	Travel history outside Brunei Local community transmission
Wang et al. [47]	China (Wuhan)	05/01/2020 to 12/02/2020	Wuhan Union Hospital	35	Median= 37 Range(25–88)	0.59	27 health care worker 10 relatives to health care worker
Viego et al. [33]	Argentina (Bahia Blanca)	20/03/2020 to 08/05/2020	Local health authorities	36	-	-	Travel history outside city Local community transmission
Tindale et al. [52]	Singapore	23/01/2020 to 26/02/2020	Publicly available data	93	-		-
	China (Tianjin)	21/01/2020 to 22/02/2020		135	-		
Tan et al. [48]	Singapore	23/01/2020 to 02/04/2020	Hospital records	164	Mean = 44.2 ±15.8	0.88	Travel history Local community transmission
Ryu et al. [34]	South Korea	20/01/2020 to 21/04/2020	Local public health authorities	2023	Median= 42 Range(1–102)	0.24	Clusters Imported from Daegu-Gyeongsangbuk or abroad Local transmission
Qin et al. [35]	China (outside Hubei) Other countries	as of 15 February 2020	Publicly available data in China The ministries of health in other countries	1084	Mean = 41.31 Median= 40	1.31	-
Nie et al. [60]	China (outside Hubei)	19/01/2020 to 08/02/2020	The website of the National Health Commission of the People’s Republic of China The health commission website of each province or city.	7015	Mean = 44.24 Range (2 m–97 y)	1.11	Resident or travelers to Wuhan Close contact with confirmed cases
Lou et al. [50]	China	19/01/2020 to 09/02/2020	Hospital records	80	Median=55 IQR [45-64]	1.58	-
Liu et al. [62]	China (Shenzhen)	04/01/2020 to 05/02/2020		365	Median=46 Range (1–86)	0.99	Contact with confirmed case-patients Wuhan Cities other than Wuhan in Hubei Province No definite exposure
Li et al. [49]	China (Shenzhen)	21/01/2020 to 09/02/2020	Hospital records	74	Mean = 44.26	0.89	Travelers to Wuhan Family clusters Sporadic cases
Lee et al. [51]	South Korea	20/02/2020 to 03/03/2020	Publicly available data	47	Median=30 Range (17–83)	-	-
Chun et al. [9]	South Korea	23/01/2020 to 31/03/2020	Public reports of confirmed COVID-19 patients by the government and each municipal website in South Korea	72	Median=40 IQR [24-54]	0.89	Contact with confirmed cases
Alsofayan et al. [53]	Saudi Arabia	01/03/2020 to 31/03/2020	Health Electronic Surveillance Network (HESN) Database	1519	Median=36	1.18	History of recent travel outside KSA Local community transmission
Bohmer et al. [42]	Germany (Bavaria)	27/01/2020 to 16/02/2020	Bavarian Health and Food Safety Authority and national level (Robert Koch Institute) public health authorities and four public health laboratories.	16	Median=35 IQR [27-42] Range (2–58)	3.0	Cluster
You et al. [36]	China (outside Hubei province)	01/01/2020 to 31/03/2020	The National Health Commission (NHC) of China	169	-	-	Resident or travelers to Hubei
Wieland [37]	Germany	15/02/2020 to 31/03/2020	Official German case data	107	-	-	-

*IQR* interquartile range, *95% CI* 95% confidence interval, *±* standard deviation

^┼^Proportion of cases on which was calculated the incubation period among all participants in the study

^┼┼^Age expressed by median [IQR] or range (x–x) or mean ±SD (years)

### Risk of bias within studies

All the 42 observational studies had the third level of evidence (non-analytic studies) with a grading D of recommendation.

**Fig. 2:**
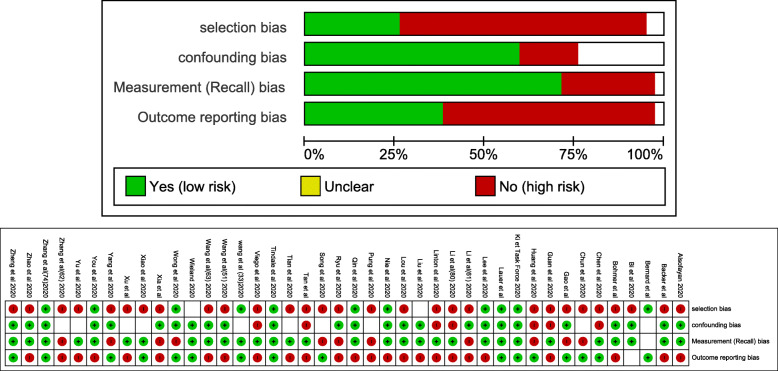
Overall and detailed risk of bias assessment among the 42 studies

Most of study had the risk of recall bias (Fig. 2).

### Results of individual studies

#### Based on studies calculating incubation period for SARS-CoV-2

The median incubation period was calculated in 17 studies ranging from 2 to 12 days with an IQR lower bound of 2 days and higher bound of 14 days. In 9 studies, the mean was ranging from 3.9 to 8.98 days.

The total incubation period ranged in 9 studies from 0 to 26 days. One study was restricted to pediatric patients infected with SARS-CoV-2 from 7 months to 17 years old. The average incubation period was 8 days ranged from 1 day to 13 days [43] (Table 2).

**Table 2:** Overview of studies calculating incubation period for SARS-CoV-2

Authors	(***n***/***N***)^┼^	Incubation period (days)		
		Median [IQR]	Mean±SD	Range
Guan et al. [27]	291/1099	4 [2.0–7.0]	-	-
Ki and Task Force for 2019-nCoV [28]	10/28	3	3.9	0–15
Chen et al. [43]	12/ 12	-	8	1–13
Gao et al. [7]	6/15	10	-	3–12
Huang et al. [8]	6/8	2.0	-	1–4
Pung et al. [38]	19/36	4 [3.0–6.0]	-	-
Song et al. [39]	22/24	-	-	2–13
Tian et al. [44]	262/262	-	6.7 ±5.2	-
Wang et al. [25]	631/631	-		Max 19
Xia et al. [40]	9/10	-	7 ± 2.59	2–14
Xu et al. [54] Imported Secondary Tertiary	15/51 17/51 19/51	8 [4.0–10.0] 8 [4.0–11.0] 12 [9.0–14.0]	-	-
Bernard et al. [41]	3/3	-	-	3–7
Yu et al. [30]	132/333 G1 (*n*=64) G2 (*n*=57) G3 (*n*=11)	7.8 [5.0–8.2] 7.5 [5.0–7.9] 9 [5.0–8.0]	- - -	0.5–20 0.5–23 1–14
Zheng et al. [45]	61/73	-	-	Max 26
Zhao et al. [46]	6/136	6 [4.0–11.0]	-	1–21
Zhang et al. [59]	194	-	7.44	0.08–18
Xiao et al. [31]	2555/ 4741	-	8.98	-
Wong et al. [32]	135/135	5.0	-	1–11
Tan et al. [48]	164	5.0	5.7±3.5	1–17
Nie et al. [60]	2907/7015	5.0 [2.0–8.0]	-	Max 24
Lou et al. [50]	45/80	5.0 [2.0–10.0]	-	0–23
Liu et al. [62]	58/365	5.0 [3.0–8.0]	6.0	1–16
Li et al. [49]	74/74	5.0 [4.0–7.0]	-	-
Alsofayan et al. [53]	309/1519	6.0 [7.5]	-	-
Bohmer et al. [42]	16	4.0 [2.3–4.3]	-	-
You et al. [36]	169/198	7.0 [4.5–10]	8.0±4.75	0–23.5

^┼^Proportion of cases on which was calculated the incubation period among all participants in the study

*SD* standard deviation, *IQR* interquartile range

### Based on studies estimating incubation periods for SARS-CoV-2

The log-normal distribution was the best fitting to the data in 7 studies with an estimated mean ranging from 5.0 to 7.4 (95% CI, 2 to 20 days) (Table 3). The median was estimated in 9 studies and had a maximum value of 7.2 (95% CI, 6.4 to 7.9 days) [58]. The estimated 95th percentile of the distribution had a maximum value of 16.32 days. The maximum 97.5th and 99th percentile of the distribution was 11.5 days and 20.4 days respectively (Table 3).

**Table 3:** Overview of studies estimating incubation periods for SARS-CoV-2

Study	*n*	Distribution	Mean (days)		95th percentile(days)	
			Estimate	95% CI	Estimate	95%CI
Li et al. [20]	10	Log normal	5.2	4.1–7.0	12.5	9.2–18
Zhang et al. [23]	49	Log normal	5·2	1.8–12.4	10.5	
Linton et al. [29]	52^€^	Log normal*	5.0 5.6 ^┼^	4.2–6.0 4.4–7.4	10.6 12.3	8.5–14.1 9.1–19.8
		Weibull	5.4	4.3–6.6	12.0	9.8–15.6
		Gamma	5.3	4.3–6.6	11.3	9.2–14.5
	158^€ €^	Log normal*	5.6	5.0–6.3	10.8	9.3–12.9
		Weibull	5.8	5.2–6.5	11.0	9.6–12.9
		Gamma	6.0	5.3–6.7	11.7	10.3–13.4
Backer et al. [55]	88	Weibull*	6.4	5.6–7.7	10.3	8.6–14.1
		Gamma	6.5	5.6–7.9	11.3	9.1–15.7
Lauer et al. [56]	181	Log normal*	5.5 5.1 ^a^	- 4.5–5.8	11.5^b^	8.2–15.6
Wang et al. [57]	483	Log normal*	7.4	2–20	-	-
Bi et al. [24]	183	Log normal	4.8^a^	4.2–5.4	14.0	12.2–15.9
Yu et al. [30]	132	Gamma	7.2^a^	6.4 -7.9	16.0 20.4 ^c^	- -
Yang et al. [26]	178	Weibull*	6 ^a^	-	13.7	12.5–14.9
Wang et al. [47]	14	Log normal	4.5	3.0–6.4	11.4	4.0–12.0
Viego et al. [33]	12	Log normal	7.50	4.11–10.89	-	-
Tindale et al. [52]	93	Gamma	5.99	4.97–7.14	-	-
	135	Gamma	8.68	7.72–9.7	-	-
Ryu et al. [34]	181	Log normal*	4.7 ^a^	0.1–15.6	-	-
Qin et al(69)	1084	Weibull	8.29	7.67–8.90	16.32	15.62-17.04
Lee et al. [51]	47	Log normal*	3.0 ^a^	0.6–8.2	-	-
Chun et al. [9]	74	Weibull	3.10 ^a^	2.54–3.71	-	-
		Gamma	2.99 ^a^	2.44–3.60	-	-
		Log normal*	2.87 ^a^	2.33–3.50	-	-
Wieland [37]	107	-	-	-	5.6 ^b^	-

^€^Excluding Wuhan resident

^€€^Including Wuhan resident

*Best fit distribution to the data

^┼^Estimation with right truncation

^a^Median

^b^97.5th percentile

^c^99th percentile

### Mean incubation period and meta-analysis

**Fig. 3:**
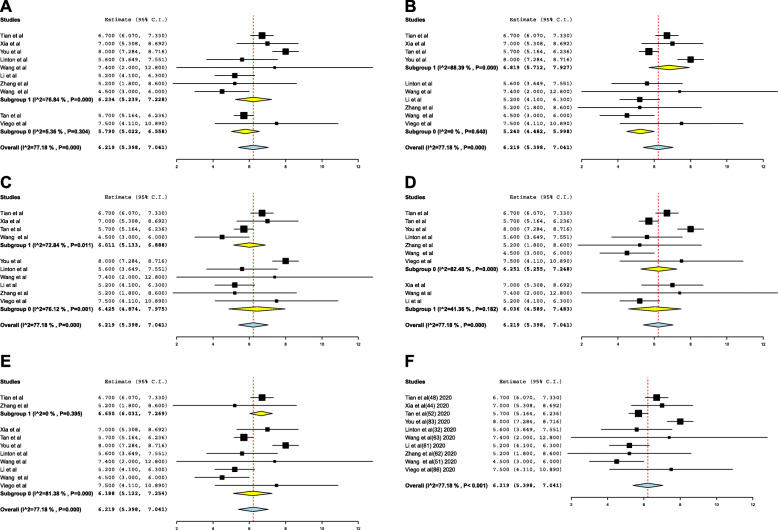
Forest-plot for mean incubation period in days

The estimated mean incubation period obtained from the included studies and the pooled mean are presented in Fig. 3. Out of the 10 included studies in the meta-analysis, 8 were conducted in China, 1 in Singapore, and 1 in Argentina. The pooled mean incubation period was 6.2 (95% CI 5.4, 7.0) days. Heterogeneity testing (*I*^2^ = 77.1%; *p* < 0.001) revealed notable differences among the included studies in the meta-analysis.

Moderator variables were analyzed to identify and eliminate the observed heterogeneity: population of studies, severity, sex-ratio, study quality, and method of calculation (Table 4).

**Table 4:** Estimation of days of incubation with moderator variables

	Estimate	SE	95% CI	*p*-value
Intercept	6.219	0.419	(5.398; 7.041)	< 0.001
Population				
Chinese	6.234	0.507	(5.239; 7.228)	< 0.001
Not Chinese	5.790	0.392	(5.022; 6.558)	< 0.001
Severity				
Hospitalized	6.011	0.448	(5.133; 6.888)	< 0.001
Not hospitalized	6.425	0.791	(4.874; 7.975)	< 0.001
Sex ratio				
>1	6.036	0.738	(4.589; 7.483)	< 0.001
<1	5.805	0.435	(4.952; 6.659)	< 0.001
Quality of study				
Strong	6.650	0.316	(6.031; 7.269)	< 0.001
Moderate to weak	6.188	0.419	(5.122; 7.254)	< 0.001

*CI* confidence interval, *SE* standard error

The heterogeneity was decreased when we included the study quality and the method of calculation used as moderator variables (*I*^2^ 0%). The mean incubation period ranged from 5.2 (95% CI 4.4 to 5.9) to 6.65 days (95% CI 6.0 to 7.2) (Fig. 3).

## Discussion

This review includes 42 studies done predominantly in China showing a mean and median incubation period of maximum 8 days and 12 days respectively. The pooled mean incubation period for COVID-19 is 6.2 (95% CI 5.4, 7.0) and may vary depending on population of studies, severity, sex-ratio, study quality, and method of calculation. In various parametric models, the 95th percentiles were in the range 10.3–16 days, which was not consistent with the first WHO reports [61]. While it was difficult to estimate the right hand tail of the incubation period distribution based on small sample sizes, the highest 99th percentile would be as long as 20.4 days, and this indicates that long incubation periods are possible. Lauer et al. [56] estimated that 101 out of 10,000 cases (99th percentile=482) would develop symptoms after 14 days of active monitoring or quarantine. Wang et al. [57] reported that about 7.45% patients were overestimated with longer than 14 days of incubation periods. Although many studies did not match with the inclusion criteria in our review, they are worthy to be mentioned. In a research letter studying serial cases of 6 patients infected with SARS-CoV-2 in China, Bai et al. reported that the incubation period of patient 1 was 19 days [63]. Based on 175 case details reported by 64 web pages between January20, 2020, and February 12, 2020, Leung estimated a mean of 7.2 (95% CI 6.1 to 8.4) with a 95th percentile of the Weibull distribution of 14.6 days (95% CI, 12.1 to 17.1) [64]. On the other hand, in the beginning of the pandemic of COVID-19, some studies found that there is no observable difference between the incubation time for SARS-CoV-2, severe acute respiratory syndrome coronavirus (SARS-CoV), and Middle East respiratory syndrome coronavirus (MERS-CoV) [11].

In our results, studies with contact tracing or exposure history of close contact showed a range of incubation period not exceeding 14 days [8, 38, 40, 41]. In fact, potential direct transmission could be related to a higher infecting dose and higher virulence of the strain that could lead to a shorter incubation period [65]. Indeed, Yu et al. showed that the incubation period was significantly shorter among patients who had multiple exposures to confirmed cases in the same province (Shanghai) (median 7.5 days; interquartile range (IQR) 5–7.9 days) compared with patients who had travel history in Wuhan (median 7.8days; (IQR) 5–8.2days) [30]. These results strengthen the hypothesis that a higher infecting dose could have been transmitted by the index case leading to a shorter incubation period compared with cases associated with “indirect” transmission.

In our study, there was considerable heterogeneity investigated with subgroup analysis. Several articles have shown that the incubation period differs between individuals according to their age or sex. Tan et al. showed that age-specific mean incubation periods were statistically significantly different across different age categories. The longest was observed among those aged 70+ (7.56 days, 95% CI 5.31–9.80) while the shortest was among those aged 60–69 years (4.69 days, 95% CI 3.86–5.52) and <30 years (4.95 days, 95% CI 4.31–5.58) [48]. However, Qin et al. concluded that there is no evidence that the incubation time depends on age [35].

Systematic reviews and meta-analyses conducted from 1 December 2019 to 11 March showed that the pooled incubation period mean was 5.68 (99% CI: 4.78, 6.59) days with heterogeneity testing (*I*^2^ = 98.4%) [66]. As in our findings, this heterogeneity test revealed notable differences among the included studies.

On the other hand, and based on the log-normal distribution, McAloon et al. [67] found in a meta-analysis conducted from December 1, 2019, to April 8, 2020, a mean of 5.8 days (95% CI 5.0–6.7) for the corresponding incubation period. However, our results with an estimated incubation mean of 5.2 (95% CI, 4.4–5.9) were more reliable since the heterogeneity test was zero.

Our study has some notable limitations. First, in most studies, the data were collected retrospectively, resulting in a recall bias (uncertain exact dates of exposure) and some missing data that would inevitably influence our assessment. Second, due to urgent timeline for data extraction and analysis, many studies have estimated the incubation period in a limited case number in a short period of time, which necessitates the cautious interpretation of the generalizability of our findings. The numbers were too small to detect systematic differences in incubation time with age or sex. Third, not all studies (except Tian et al. [44]) paid attention to asymptomatic patients, so our review may represent an erroneous incubation period. Although there is interest on asymptomatic transmission, we were unable to address this point in our review, and further studies should be done to better understand disease transmissibility of asymptomatic cases.

## Conclusions

This work provides additional evidence of incubation period for COVID-19 and showed that it is prudent not to dismiss the possibility of incubation periods up to 14 days at this stage of the epidemic. As the epidemic continues, it remains important to collect more information on incubation periods through longitudinal studies with more patients so that we can conduct subgroup analysis and better understand the transmissibility of.

## Supplementary Information

**Additional file 1:.** Quality assessment of the included studies.

## Data Availability

Not applicable

## Abbreviations

EPHPP: Effective Public Health Practice Project
MERS-CoV: Middle East respiratory syndrome coronavirus
IQR: Interquartile range
RCTs: Randomized controlled trials
SARS-CoV: Severe acute respiratory syndrome coronavirus
SD: Standard deviation
SIGN: Scottish Intercollegiate Guidelines Network
95% CI: 95% confidence interval
