# Supplementary material for: The incubation period during the pandemic of COVID-19: a systematic review and meta-analysis
Source: Syst Rev. 2021 Apr 8;10:101. doi: 10.1186/s13643-021-01648-y (PMC8031340; doi:10.1186/s13643-021-01648-y)
Supplement: Supplementary file 1 — Additional file 1:. Quality assessment of the included studies. [file 13643_2021_1648_MOESM1_ESM.docx]

Appendix1: Quality assessment of the included studies

| Authors | Selection Bias | Study Design | Data Collection Method | Global Rating For each Paper |
| --- | --- | --- | --- | --- |
| Guan et al(30) | Q1:2  Q2:4  Moderate | 7: DCSS  Weak | Q1:1  Q2:1  Strong | MODERATE |
| Li et al(61) | Q1:3  Q2:4  Weak | 5:DLS (contact tracing)  Moderate | Q1:1  Q2:1  Strong | MODERATE |
| Ki et Task Force(31) | Q1:2  Q2:4  Moderate | 7 : Descriptive serial cases  Weak | Q1:1  Q2:2  Moderate | MODERATE |
| Linton et al(32) | Q1:2  Q2:4  Moderate | 7: DCSS  Weak | Q1:1  Q2:1  Strong | MODERATE |
| Backer et al(84) | Q1:3  Q2:4  Weak | 7 :DCSS  Weak | Q1:2  Q2:2  Weak | WEAK |
| Chen et al(47) | Q1:3  Q2:4  Weak | 7: DCSS  Weak | Q1:1  Q2:1  Strong | WEAK |
| Gao et al (72) | Q1:3  Q2:4  Weak | 5: DLS (cluster)  Moderate | Q1:1  Q2:1  Strong | MODERATE |
| Huang et al(8) | Q1:3  Q2:4  Weak | 5: DLS (contact tracing)  Moderate | Q1:1  Q2:1  Strong | MODERATE |
| Pung et al(42) | Q1:2  Q2:5  Moderate | 5: DLS (contact tracing)  Moderate | Q1:1  Q2:1  Strong | STRONG |
| Lauer et al(65) | Q1:2  Q2:5  Moderate | 7: DCSS  Weak | Q1:1  Q2:1  Strong | MODERATE |
| Song et al(43) | Q1:2  Q2:5  Moderate | 5: DLS (contact tracing)  Moderate | Q1:1  Q2:2  Moderate | STRONG |
| Tian et al(48) | Q1:2  Q2:5  Moderate | 7: DCSS  Weak | Q1:1  Q2:1  Strong | MODERATE |
| Wang et al(33) | Q1:2  Q2:5  Moderate | 7 : (ecological study)  Weak | Q1:2  Q2:2  Weak | WEAK |
| Wang et al(63) | Q1:2  Q2:5  Moderate | 7: DCSS  Weak | Q1:2  Q2:2  Weak | WEAK |
| Xia et al(44) | Q1:2  Q2:4  Moderate | 5: Interrupted time series  Moderate | Q1:1  Q2:1  Moderate | STRONG |
| Xu et al(73) | Q1:2  Q2:5  Moderate | 7: DCSS    Weak | Q1:1  Q2:2  Moderate | MODERATE |
| Zhang et al(62) | Q1:1  Q2:5  Strong | 5: DLS (contact tracing)  Moderate | Q1:1  Q2:1  Strong | STRONG |
| Bernard et al(45) | Q1;3  Q2:3  Weak | 7: Descriptive Serial cases  Weak | Q1:1  Q2:2  Moderate | MODERATE |
| Bi et al(26) | Q1;2  Q2:3  Moderate | 5: DLS (contact tracing)  Moderate | Q1:1  Q2:2  Moderate | STRONG |
| Yu et al(34) | Q1;2  Q2:3  Moderate | 7: DCSS  Weak | Q1:1  Q2:2  Moderate | MODERATE |
| Zheng et al(49) | Q1:2  Q2:5  Moderate | 7 : DCSS  Weak | Q1:2  Q2:2  Weak | WEAK |
| Zhao et al(50) | Q1:2  Q2:5  Moderate | 7 : DCSS  Weak | Q1:2  Q2:2  Weak | WEAK |
| Zhang et al(74) | Q1:2  Q2:5  Moderate | 7 : DCSS  Weak | Q1:2  Q2:2  Weak | WEAK |
| Yang et al(85) | Q1;2  Q2:3  Moderate | 5: DLS (contact tracing)  Moderate | Q1:1  Q2:2  Moderate | STRONG |
| Xiao et al(75) | Q1;2  Q2:3  Moderate | 7: DCSS  Weak | Q1:1  Q2:2  Moderate | MODERATE |
| Wong et al(76) | Q1;2  Q2:3  Moderate | 5: DLS (contact tracing)  Moderate | Q1:1  Q2:2  Moderate | STRONG |
| Wang et al(51) | Q1;2  Q2:3  Moderate | 7: DCSS  Weak | Q1:1  Q2:2  Moderate | MODERATE |
| Viego et al(86) | Q1:2  Q2:4  Moderate | 7: DCSS  Weak | Q1:1  Q2:1  Strong | MODERATE |
| Tindale et al(87) | Q1:3  Q2:4  Weak | 7: DCSS  Weak | Q1:2  Q2:2  Weak | WEAK |
| Tan et al(52) | Q1;2  Q2:3  Moderate | 7: DCSS  Weak | Q1:1  Q2:2  Moderate | MODERATE |
| Ryu et al(88) | Q1:2  Q2:4  Moderate | 5: Interrupted time series  Moderate | Q1:1  Q2:1  Moderate | STRONG |
| Qin et al(69) | Q1:3  Q2:4  Weak | 7 :DCSS  Weak | Q1:2  Q2:2  Weak | WEAK |
| Nie et al(77) | Q1:3  Q2:4  Weak | 7 :DCSS  Weak | Q1:2  Q2:2  Weak | WEAK |
| Lou et al(78) | Q1:2  Q2:4  Moderate | 7: DCSS  Weak | Q1:1  Q2:1  Strong | MODERATE |
| Liu et al(79) | Q1:3  Q2:4  Weak | 7 :DCSS  Weak | Q1:2  Q2:2  Weak | WEAK |
| Li et al(80) | Q1:3  Q2:4  Weak | 7: DCSS  Weak | Q1:1  Q2:2  Moderate | MODERATE |
| Lee et al(89) | Q1:2  Q2:4  Moderate | 7 :DCSS  Weak | Q1:2  Q2:2  Weak | WEAK |
| Chun et al(9) | Q1;2  Q2:3  Moderate | 5: DLS (contact tracing)  Moderate | Q1:1  Q2:2  Moderate | STRONG |
| Alsofayan(81) | Q1;2  Q2:3  Moderate | 7: DCSS  Weak | Q1:1  Q2:2  Moderate | MODERATE |
| Bohmer et al(82) | Q1;2  Q2:3  Moderate | 7: Serial cases  Weak | Q1:1  Q2:2  Moderate | MODERATE |
| You et al(83) | Q1:3  Q2:4  Weak | 7 :DCSS  Weak | Q1:2  Q2:2  Weak | WEAK |
| Weiland(90) | Q1:3  Q2:4  Weak | 7 :DCSS  Weak | Q1:2  Q2:2  Weak | WEAK |

DCSS: Descriptive Cross Sectional Study/ DLS: Descriptive longitudinal study
